# Super-Resolution Imaging of Bacteria in a Microfluidics Device

**DOI:** 10.1371/journal.pone.0076268

**Published:** 2013-10-16

**Authors:** Diego I. Cattoni, Jean-Bernard Fiche, Alessandro Valeri, Tâm Mignot, Marcelo Nöllmann

**Affiliations:** 1 Centre de Biochimie Structurale, Centre National de la Recherche Scientifique, Unité Mixte de Recherche 5048, Montpellier, France; 2 Institut Nationale de la Santé et la Recherche Médicale, Unité 1054, Montpellier, France; 3 Universités Montpellier I et II, Montpellier, France; 4 Laboratoire de Chimie Bactérienne, Centre National de la Recherche Scientifique, Aix-Marseille University, Unité Mixte de Recherche 7283, Marseille, France; University of Groningen, Groningen Institute for Biomolecular Sciences and Biotechnology, The Netherlands

## Abstract

Bacteria have evolved complex, highly-coordinated, multi-component cellular engines to achieve high degrees of efficiency, accuracy, adaptability, and redundancy. Super-resolution fluorescence microscopy methods are ideally suited to investigate the internal composition, architecture, and dynamics of molecular machines and large cellular complexes. These techniques require the long-term stability of samples, high signal-to-noise-ratios, low chromatic aberrations and surface flatness, conditions difficult to meet with traditional immobilization methods. We present a method in which cells are functionalized to a microfluidics device and fluorophores are injected and imaged sequentially. This method has several advantages, as it permits the long-term immobilization of cells and proper correction of drift, avoids chromatic aberrations caused by the use of different filter sets, and allows for the flat immobilization of cells on the surface. In addition, we show that different surface chemistries can be used to image bacteria at different time-scales, and we introduce an automated cell detection and image analysis procedure that can be used to obtain cell-to-cell, single-molecule localization and dynamic heterogeneity as well as average properties at the super-resolution level.

## Introduction

Bacteria have evolved complex, highly-coordinated, multi-component cellular engines, such as the apparatus responsible for chromosome segregation/cell division/separation, the flagellar motor, the transcription/replication machines, or secretion/conjugation machineries, to achieve high degrees of efficiency, accuracy, adaptability, and redundancy [1]. Studying the cellular localization, composition, dynamics and architecture of these molecular machines is key for understanding their function and mechanism. Conventional fluorescence microscopy methods enable non-invasive observation of protein organization and localization in live cells with high specificity, and have played an important role in the investigation of these processes. However, the maximum resolution attainable by these methods is intrinsically limited by light diffraction and is several orders of magnitude lower than for X-ray or electron tomography. This limitation is considerably acute for bacteria, as the maximal resolution (∼250 nm) is comparable to the size of the cell (typically ∼1–2 um). As a result, the structures and dynamics of important bacterial machineries, often smaller than the diffraction limit, could not be directly probed *in vivo*. Recent advances in fluorescence microscopy have led to the development of several conceptually independent super-resolution methods that break the intrinsic resolution limit imposed by the diffraction of light [2], [3]. In particular, single-molecule based super-resolution microscopy (smSRM) methods, such as photo-activated localization microscopy (PALM) and stochastic optical reconstruction microscopy (STORM) or other variants based on the same principles [4], [5], , have the advantage that they reach the highest resolutions (20–40 nm) and are applicable to live cells.

smSRM relies on the stochastic photo-activation and single-molecule localization of individual probes in order to reconstruct an image from the coordinates of thousands of localizations. The acquisition of a smSRM image: (1) is intrinsically slow (typically 1–40 min), as it requires a density of localization low enough so that a single emitter is activated at any given time in any diffraction-limited area; (2) necessitates a means for correcting sample drift with nanometer precision, which typically involves the use of fiducial marks; (3) needs a high signal-to-noise ratio of detection and low background to achieve high localization precision [9]. Up until now, most fluorescence microscopy experiments of bacterial processes used thin agarose pads to provide a substratum that supports growth or surface motility [10], [11], [12], [13], [14], PDMS-based microfluidics chambers to physically immobilize cells [15], [16], [17], or hybrid microfluidics devices in which thin agarose pads are combined with a custom flow chamber [18], [19], [20], [21]. To date, several super-resolution studies in bacteria have used these methodologies [22], [23], [24], [25], [26] despite some important limitations, such as: (1) poor long-term stability due to drift of the agarose pad produced by desiccation and local melting or by movement of cells in micro-fluidics chambers (due in part to flow); (2) increased background levels; (3) lack of surface flatness; (4) and fluorescence signal bleed-through between channels in multi-color experiments.

Here, we present a method in which cells are functionalized to a microfluidics device and fluorophores are injected and imaged sequentially. This method has several advantages, as it permits the long-term immobilization of cells and proper correction of drift, avoids chromatic aberrations caused by the use of different filter sets, and allow for the flat immobilization of cells on the surface. In addition, we show that different surface chemistries can be used to image at different time-scales, and develop an automated cell detection and image analysis procedure that can be used to obtain cell-to-cell, single-molecule localization and dynamic heterogeneity as well as average properties at the super-resolution level.

## Results

### PALM experiments on agarose pads

A typical PALM experiment requires the acquisition of thousands of sequential images for considerably long timescales (∼1–40 min) until all photo-activatable (PA) proteins have been localized and photo-bleached. To be able to reconstruct a proper PALM image, it is of utmost importance that cells remain immobile during the whole acquisition time. This has been ordinarily achieved by immobilizing cells in agarose pads, which have the added advantage that they allow for time-lapse imaging under conditions that promote normal growth [13], [14], [27], [28].

**Figure 1:**
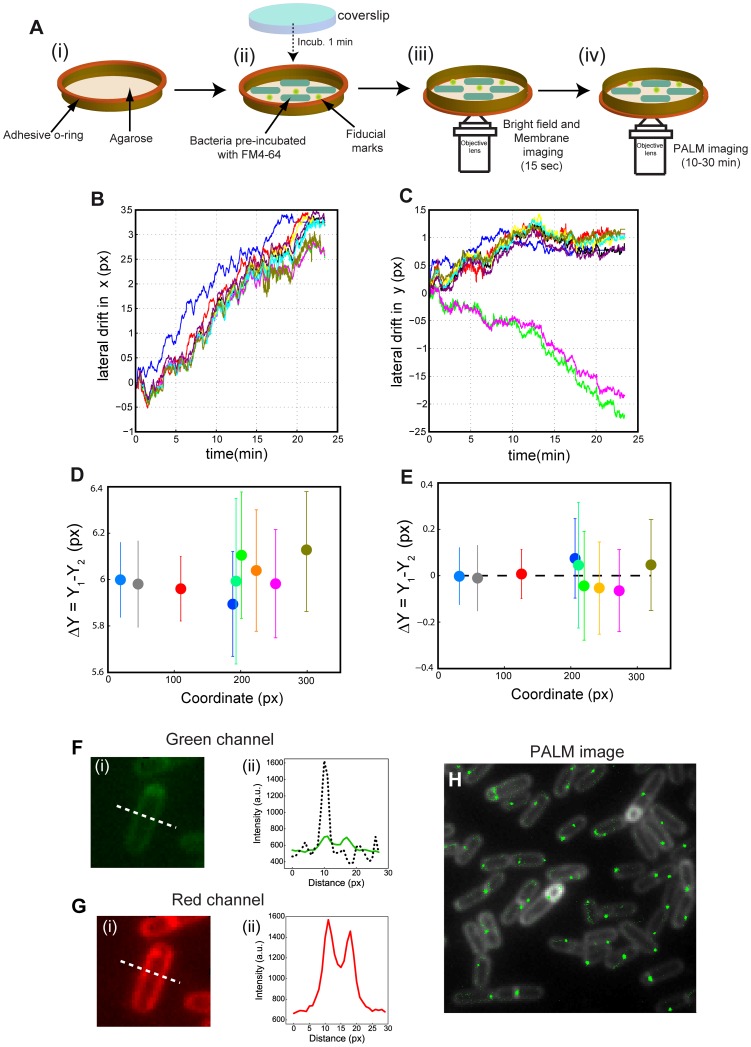
smSRM of bacteria in agarose pads. ** A. smSRM imaging of bacteria in agarose pads.** (i) A double-side adhesive o-ring was placed on a coverslip and melted agarose was added to create an adhering surface for the bacteria. (ii) Bacterial cells, previously stained with the membrane dye FM4-64 mixed with fiducial marks, were deposited on agarose and the pad was sealed with a clean coverslip. The sample was finally fixed on an Attofluor cell (Invitrogen) to avoid bacterial motion during microscopy. (iii–iv) Sequential imaging of bacterial membrane and SpoIIIE (iii) Epi-fluorescence image of the cell membrane was collected by exiting at 532 nm. (iv) smSRM images were collected by using continuous excitation with a 532 nm laser and by applying regular pulses of photo-activation with a 405 nm laser. **B–C. Lateral drift during smSRM acquisition in agarose pads.** Lateral drift over the full acquisition period was assessed by plotting the trajectories of fluorescent beads in *x* (B) and *y* (C) coordinates over time. Each colored trajectory corresponds to a single fluorescent bead. **D–E. Alignment correction in smSRM experiments in agarose pads.** Distortion arising from chromatic aberrations was quantified from the distance between the same fluorescent beads observed in two different emission channels (D) and corrected by using a linear transformation procedure (E) (see Materials and Methods). Each dot represents a different bead and the abcissa represents the *x* coordinate of each bead. Error bars represent the precision of localization before (D) and after (E) drift and alignment correction. **F–G. Bleed-through of the membrane staining agent FM4-64 during smSRM imaging in agarose pads. (i)** Image of a cell in the SpoIIIE-PA (SpoIIIE-eosFP) (F) and FM4-64 (G) channels. (ii) Line scans of the fluorescence signal across a *B. subtilis* cell (white dotted line in panels F-i and G-i) in the two observation channels (green and red lines, respectively). For comparison, the line scan of the fluorescence intensity emitted by a single SpoIIIE-PA protein was overlapped in F-ii (black dotted line). As expected, the signal-to-noise ratio and contrast in the red channel are adequate (SNR = 40/contrast = 2.3, panel G-ii). However, even at low dye concentrations the fluorescence signal from FM4-64 bleeds into the SpoIIIE-PA channel (SNR = 8/contrast = 1.3, panel F-ii), compromising single-molecule detection, lowering the localization precision, and often leading to false positive localizations. For comparison, in the single-molecule trace shown in F-ii the signal to noise ratio is 30, and the contrast is 3. **H. SpoIIIE localization observed by smSRM in agarose pads.** Pointillist representation of SpoIIIE-PA localization in *B. subtilis* at different cell stages. Each green dot represents a single fluorescent event detected in a single frame during the smSRM acquisition. False positive localizations can be observed scattered homogeneously over the cell membrane.

To test the limits of agarose pad immobilization, we performed smSRM experiments on a *B. subtilis* strain carrying a fusion of the DNA translocase SpoIIIE [29] to the photo-activatable fluorescent proteins mMaple [30] or eosFP. Sporulating cells were stained with the membrane stain FM4-64, immobilized on an agarose pad, and fluorescent beads were added to serve as fiducial marks (Figure 1A-ii, and Materials and Methods). A field of view containing tens of cells was first imaged by bright-field microscopy, then the cell contour was imaged by detecting the fluorescence signal emitted by FM4-64 (Figure 1A-iii), and finally a complete PALM dataset comprising of ∼20000 frames was acquired (Figure 1A-iv). These acquisitions were performed sequentially using excitation and emission wavelengths adapted to each fluorophore (see Materials and Methods). Acquisition of a PALM dataset involved continuous excitation with a readout laser (at 561 and 532 nm, 0.5–1 kW/cm^2^) and short, regularly-spaced pulses of photo-activation with a UV laser (at 405 nm, 10 W/cm^2^). These values were optimized for the detection of single photo-activatable proteins while preventing activation induced by the read-out laser and cell photo-damage. Typically, high excitation powers are used to increase the photon count and thus improve the localization precision [9], and the position of at least seven fiducial marks are used to correct for chromatic aberrations and long-term lateral drift of the sample during acquisition (Materials and methods). This methodology allows for the reconstruction of PALM images, but closer inspection of the acquisition dataset reveals several drawbacks.

First, we quantified the movement at the nano-metric scale of different, spatially-separated fiducial marks in the same field of view. The movement was quantified by monitoring the long-term drift in the x and y directions observed for different fiducial marks as a function of time (Figure 1B–C). We observed a typical mean drift of ∼200 nm over a time period of ∼10 min (Figure 1B–C). Additionally, in an important percentage of experiments, beads in different locations in the field-of-view showed different trajectories of fiducial marks in the *x* and *y* directions (see blue, green and magenta traces in Figure 1B–C). This behavior indicates that in agarose pads lateral movement of fiducial marks is not only associated with long-term drift of the chamber, but also to the agarose support itself. The origin of anisotropic local movements can be explained by slow local changes in the structure of the agarose matrix that affect unequally the different fiducial marks, a phenomenon that is possibly triggered by laser-induced heating and/or pad desiccation. This local melting behavior was observed even at high agarose concentrations (>1.5%) and lower laser powers (∼0.1 kW/cm^2^). This problem can be partially solved by excluding the cells in regions were a high degree of drift was observed (as the localizations on those regions cannot be properly corrected) and by drift correcting the coordinates arising from each cell by using only a subset of fiducial marks (typically 5) located as closely as possible to that cell in order to minimize the distortions introduced by local movements. This methodology is efficient but leads to a reduced resolution of localization, and to a low efficiency in data collection, as large number of cells have to be discarded. In addition, this procedure requires the presence of a large number of fiducial marks on the sample, whose strong fluorescence emission signal degrades the quality of signal collection from single-molecules.

Second, we investigated the effect of chromatic aberrations in the superposition of images taken in different fluorescence emission channels (Figure 1D). The accurate correction of distortions introduced by chromatic aberrations of the imaging system is key to properly superimpose the localizations of different proteins at super-resolution, as well as to determine precisely the localization of single molecules with respect to cellular structures such as membranes. Typically, these chromatic aberrations are corrected by sequentially imaging fiducial marks emitting in both detection channels in the same field of view, and finding the polynomial function that best transforms the set of fiducial coordinates in one color to the other. This procedure requires at least seven fiducial marks to correct for translations, rotations and expansions (see Materials and Methods). The method works well (∼15 nm correction in standard deviation, Figure 1E) in a region close to the fiducial marks (distances <5–10 μm), but tends to produce higher distortions at distances farther away probably due to the roughness and the lack of flatness of the agarose pad surface. More sophisticated procedures for chromatic aberration correction exist [31], [32], [33] but require the presence of a large density of fiducial marks on the sample, thus considerably compromising the detection of single-molecules and the throughput of the experiment.

Thirdly, cells immobilized on agarose pads are typically not flat, and may in some instances show a tilt of a few degrees that can considerably affect the measurement of relative distances between proteins and between proteins and cellular structures [34]. The depth of field (distance away from the focus that can be imaged on the camera) in smSRM imaging is characteristically reduced by the low emission of single-molecules (a rapid lose in signal away from the focal plane) and the stringent criteria utilized for localizing single-molecules, and is typically of ∼200 nm, but can vary with the brightness of the probe. Due to this reduced depth of field in smSRM, even a small tilt in the orientation of the major cell axis can lead to a dramatic change in the plane that is imaged for each cell, and to heterogeneities in protein structures between different cells as a 2D-PALM image represents the projection onto the imaging plane of the distribution of proteins within the depth of field.

Fourthly, to image the bacterial membrane in agarose pads it is necessary to incubate cells with a membrane dye prior to their deposition on agarose pads. To minimize the detection of fluorescence arising from the membrane dye (due to its often large stokes shift and broad emission spectra) during smSRM imaging, dye concentration has to be lowered significantly with respect to the concentrations typically used in standard fluorescence microscopy experiments [35], [36], leading to a less than optimal contrast, defined as the ratio of fluorescent intensities between specific and background signals (2.3 in Figure 1G, i and ii). Additionally, the membrane dye can still be detected in the smSRM channel (Figure 1F, i), and its contribution to the total fluorescence signal is not negligible (contrast of 1.3, Figure 1F, ii), and is comparable to that of single-molecule emitters (PA proteins typically show a contrast of ∼2–3 in our experimental setup). More importantly, this increased fluorescence background not only degrades the localization precision but also can lead to false positive localizations (Figure 1H). Optimization of excitation and emission wavelengths can be used to reduce the impact of bleed-through between channels, but not to eliminate it completely.

Finally, there is a number of other limitations related to the immobilization of bacterial cells in agarose pads that are common to all microscopies but more acute for live smSRM imaging: (1) in sealed agarose pads, it is difficult to renew the growth medium or to change it, for instance to test the effects of drugs or growth conditions. In the past, this problem was solved by passing a flow through a chamber in contact with the pad that allows diffusion through the agarose [18], [21]. However, in this approach only small molecules that do not interact with agarose can get to the cells and the speed at which the medium can be changed is limited by the kinetics of diffusion through the agarose pad; (2) desiccation of pads leading to major long-term drift in the lateral (discussed above) and axial directions, the latter being difficult to correct during a smSRM acquisition as the focal plane is usually maintained by feeding back on the position of the coverslip surface, not the cells themselves; (3) background fluorescence from agarose pads and increased cytoplasmatic fluorescence induced by the deposition of cells in agarose decrease the signal-to-noise ratio and thus degrades the localization precision; and (4) cells immobilized on agarose pads are typically found in close physical contact (Figure 1H). Thus, the fluorescence emission signals from membranes of neighboring cells overlap spatially and can make the automatic and accurate detection of their contour particularly difficult.

### smSRM experiments in a micro-fluidics chamber

**Figure 2:**
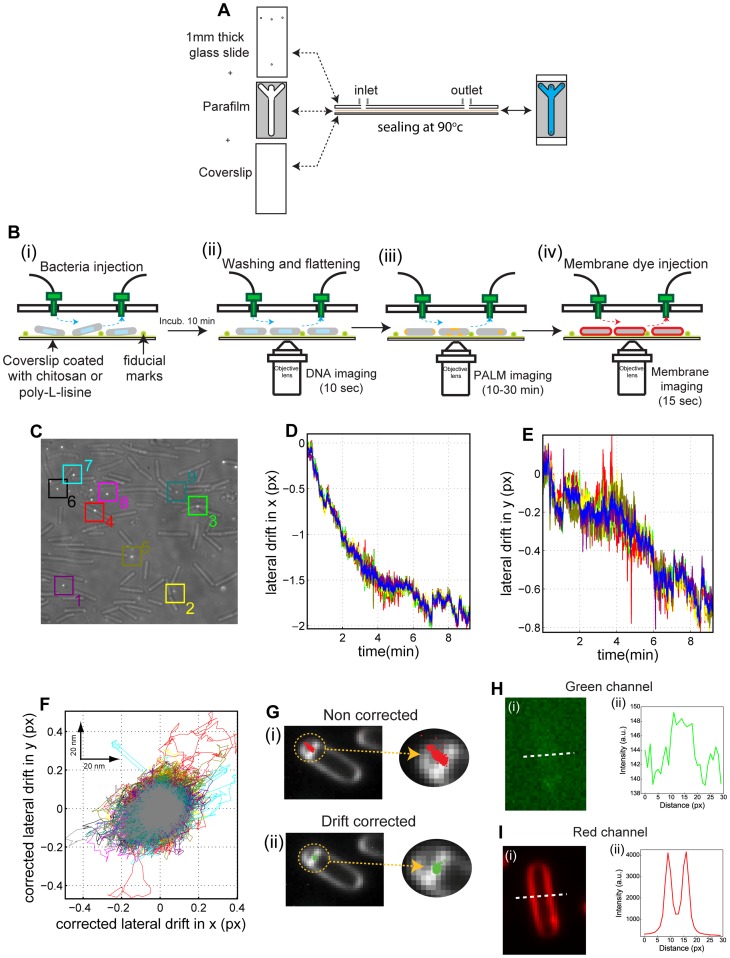
smSRM of bacteria in a microfluidics chamber. **A. Micro-fluidic chamber assembly.** A a coverslip and a 1-way inlet and single outlet ports were sealed together by a parafilm mask melted at 90 °C during 1 minute. **B. Sequential smSRM imaging procedure in microfluidic chamber.** (i) The microfluidic chamber was filled with a 0.01% (w/v) solution of poly-L-Lysine or 0.015% (w/v) chitosan and incubated for at least 5 minutes at room temperature. After washing with sporulation media, 100 μL of a concentrated solution of bacterial cells along with fiducial marks were injected and let settle onto the coated surface. (i–ii) A high flow force was applied by pumping sporulation medium to rinse the channel, wash away unattached bacteria and ensure that attached bacteria laid completely flat on the surface. (ii) DNA was imaged by epi-fluorescence microscopy, and (iii) SpoIIIE was imaged by smSRM. (iv) Finally, the FM4-64 membrane staining agent was injected allowing for bacterial membrane detection by epi-fluorescence. **C–E. Lateral drift during smSRM acquisition in micro-fluidics chambers. C.** Bright field image of *B. subtilis* in a microfluidics chamber coated with chitosan. During bright field image acquisition the 561 nm laser was turned on to simultaneously detect fiducial marks. Colored squares indicate the selected beads for drift calculation and correction. **D–E.** Lateral drift over the full acquisition period was assessed by plotting the trajectories of fluorescent beads in x (D) and y (E) coordinates over time. Each colored trajectory corresponds to a single fluorescent bead selected in C. Blue line represent the mean of all trajectories. **F. Quantification of lateral displacement after drift correction.** The lateral displacements of the different fiducial marks were recalculated after subtraction of the drift from the mean trajectory. Grey line represents the mean drift obtained for the selected group of beads (σ_x_ = 5.4 nm and σ_y_ = 6.1 nm). **G. Effect of drift correction on smSRM imaging.** Pointillist reconstruction of detected events during smSRM imaging of SpoIIIE-PA (SpoIIIE-mMaple) in sporulating *B. subtillis*. Red and green dots represent the detected events before (i) and after (ii) drift correction, respectively. The size of the dots representing single molecule detections has been artificially increased for visualization purposes. **H. Background in the SpoIIIE-PA channel during smSRM.** Line scan of the fluorescence signal across a *B. subtilis* cell in the green (SpoIIIE-PA) detection channel (white dotted line in panels H-i). The background in the green channel corresponding to cell autofluorescence is extremely low (SNR = 0.3/contrast = 1.04) as compared to that observed in the presence of low amounts of membrane dye in agarose pads (SNR = 8/contrast = 1.3) and considerably lower than the signal from single-molecules (SNR∼30/contrast ∼5–10). **I. Imaging bacterial membranes after smSRM acquisition.** Line scans of the fluorescence signal across a *B. subtilis* cell in the red channel after injection of FM4-64 (white dotted line in panels I-i). As expected, the signal-to-noise ratio and contrast in the red channel are excellent (SNR = 200/contrast = 40, panel I-ii).

To overcome these shortcomings, we developed a flexible, easy to assemble, and accessible micro-fluidics system that is ideally suited for smSRM microscopy, as it allows for the sequential imaging of fluorophores thus avoiding chromatic aberrations and channel bleed-through. This system is highly stable over long periods of time, and allows for time-lapse imaging of living cells. Fluidics chambers consisted of a ∼ 130 μm melted parafilm mask with one or several inlets and an outlet, sandwiched between a coverslip and a slide with drilled holes (Figure 2A). A thick glass slide was used to ensure a better rigidity of the chamber and to avoid deformation when approaching the objective lens. The parafilm mask and the holes in the slide were custom designed and produced by using a laser cutter (Materials and Methods). Coverslips were functionalized by using either poly-L-lysine or chitosan [37]. Cells are very efficiently adsorbed on poly-L-lysine-treated surfaces and remain immobile over long periods of time, which is ideal for smSRM experiments but deleterious for the physiology of the cell. On the other hand, cells adhered on chitosan-treated surfaces are immobile over shorter periods of time, that can be adapted to tailor the maximum degree of cell movement to the time required to perform a smSRM acquisition (which depends on the intrinsic dynamics of the imaged structure).

Prior to injection of bacteria, each channel was filled with a 0.01% (w/v) solution of poly-L-lysine or 0.015% (w/v) of chitosan, and incubated for at least 5 minutes at room temperature. A cell culture (1 ml, OD ∼0.5) containing the DNA-stain agent SYTOX was spun down, resuspended in 100 µl of filtered medium, mixed with fiducial marks, injected into the micro-fluidics chamber, and let settle on the surface for several minutes (Figure 2B-i). Next, a high flow force was applied by pumping (∼2–3 ml) of medium at ∼200 µl/s to flatten cells against the surface and rinse the channel (Figure 2B-ii). The ratio between bacteria and beads was adjusted to obtain an optimal density of 5–15 beads per field of view. In average, 100–150 bacteria per field of view could be imaged at high resolution (magnification: 150X, Figure 2C).

At this stage, chromosomes were imaged by epi-fluorescence microscopy (Figure 2B ii) and a smSRM acquisition typically consisting of 10.000–25.000 images was acquired under continuous illumination of a 561 nm read-out laser with a typical power density on the sample of 0.2 kW.cm^−^^2^ (Figure 2B-iii). This value was optimized for the detection of single photo-activatable proteins while preventing activation-induced by the read-out laser and cell photo-damage. Pulses or continuous illumination with a 405 nm laser were used to photo-activate single emitters. Pulse length and power were slowly increased during the course of the experiment in order to maintain the density of photo-activated fluorophores constant. Finally, after all fluorescent proteins have been activated and photo-bleached, 100 µl of a 10 nM membrane stain solution (FM4-64) was slowly injected in the fluidic chamber and used to image bacterial membranes (Figure 2B-iv). The three sets of images were acquired using the same four-band dichroic mirror and a single emission filter, a procedure that was only possible due to the sequential injection of dyes. This procedure completely avoided chromatic aberrations between images of different fluorophores and fluorescence bleeding that degraded the resolution of smSRM acquisitions in agarose pad experiments (see above).

Lateral drift over the full acquisition period was assessed by plotting the trajectories of fiducial marks in *x* and *y* coordinates over time. Typically, lateral sample drift can amount to hundreds of nanometers during a smSRM acquisition (Figure 2D–E). Unlike the behavior of fiducial marks in an agarose pad, marks on a microfluidics chamber are stable and follow homogeneously the same trajectory (Figure 2D–E). The quality of the drift correction was estimated by subtracting the reference to all the trajectories (Figure 2F) and calculating the standard deviations along *x* and *y*. An excellent drift correction was achieved over the whole field of view (σ = 6.5 nm for poly-L-lysine-coated surfaces and σ = 7.5 nm for chitosan-coated coverslips over a periods >10 min, Figure 2F). This procedure was used to correct the coordinates of each smSRM localization and overlay them with DNA and membrane images. An example of the quality of this procedure can be observed in Figure 2G (panel i: not corrected, panel ii: drift-corrected localizations) in which the size of SpoIIIE clusters are only limited by the localization precision (∼25 nm in our experiments). The size of these PALM-limited SpoIIIE clusters were independent of the position of the cells in the field of view, further confirming the quality of drift correction in the whole sample plane.

Under these conditions, the background fluorescence signal can be made extremely low as it only depends on the background from the media (can be optimized to almost undetectable levels) and from cells, which typically depend on growth conditions. Under similar acquisition conditions, the background level is low in microfluidics chambers (∼150 arbitrary units, Figure 2H-i and ii compared to 550 arbitrary units in agarose pads, Figure 1F-i and ii), while cell auto-fluorescence is almost indistinguishable from the support background (SNR = 0.3/contrast = 1.04, Figure 2H-i, ii). In microfluidics chambers, membrane dye is injected after smSRM data collection, thus its concentration can be adapted to obtain very high signal-to-noise ratios and contrasts (SNR = 200/contrast = 40 in Figure 2I-i, ii versus SNR∼40/contrast ∼2.3 in agarose pads, Figure 1G-i, ii), and permit an accurate reconstruction of bacterial contours and a precise localization of the center of septa during division and sporulation [34]. Under these conditions, SpoIIIE-mMaple can usually be detected with a SNR∼30 and a contrast of ∼5–10.

**Figure 3:**
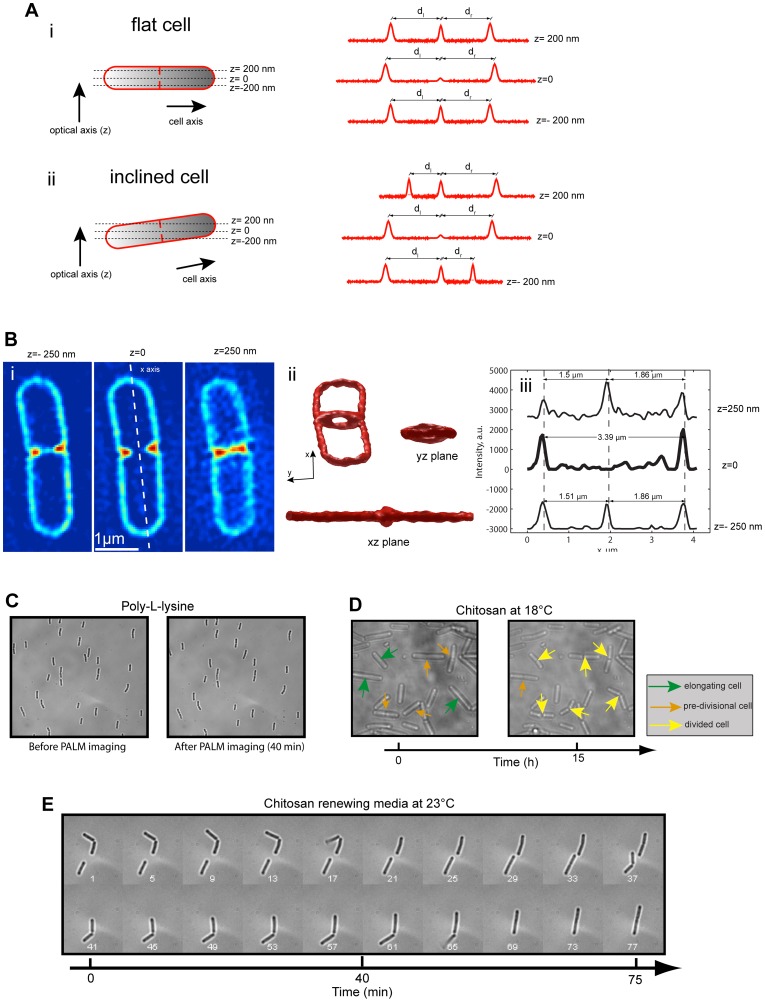
Cell flatness, stability and growth in microfluidics chambers. **A. Fluorescence intensity profiles of flat and inclined cells.** Schematic fluorescence intensity profiles are drawn across a line passing through the cell poles and the center of a closing septum at different axial positions (left panels). z represents the direction of the optical axis, with z = 0 corresponding to the plane in which the center of the septum is on focus. (i) On a flat cell, the distance between the peaks corresponding to the positions of the cell poles and the center of the cell (d_R_ and d_L_, respectively) either remain constant or diminish as the axial focal position increases or decreases. (ii) In contrast, in inclined cells the cell pole peaks move together to the left or the right as the axial position increases, and move to the opposite direction when the axial position decreases. This movement translates into an increase of d_R_ and a decrease of d_L_ on one side of the focal plane and the reverse on the opposite side. Intensity profiles are schematic and not from real data. **B. 3D-SIM membrane imaging of a dividing** ***B. subtilis*** **cell and profile quantification.** (i) Three planes of a single dividing cell are shown with the x-axis representing the axis of the cell. (ii) Three views of a constant intensity level reconstruction of the cell shown in (i). (iii) Intensity profiles drawn in the *x* direction at the three focal plane positions shown in (i). **C. Integrity and stability of** ***B. subtilis*** **during smSRM imaging on poly-L-lysine-treated surfaces.** Cells were incubated for 2 h in sporulation media, injected into a poly-L-lysine-coated microfluidics chamber, and attached to the surface as described in Figure 2B. Bright field images of sporulating *B. subtilis* cells before (left panel) and after (right panel) smSRM imaging (∼40 min). Notice that cells do not show any movement or sign of damage due the poly-L-lysine surface coating or due to smSRM imaging. Medium was continuously renewed by flow (5 µl/min of LB20%). **D–E.** ***B. subtilis*** **can grow and divide after smSRM imaging on chitosan-treated surfaces.** Exponentially growing cells were injected into a chitosan-treated microfluidics chamber, and attached to the surface as described in Figure 2B. Representative examples of *B. subtillis* spotted on chitosan and followed by time-lapse bright field imaging under two conditions: (D) at 18°C with no medium renewal, and (E) at 23°C while renewing the growing medium (LB20%). Time between frames was 8 min. Images were taken from Movie S1. Frame numbers (white) are indicated in the time-lapse montage of panel D. Color-coded arrows indicate elongating (green), pre-divisional (orange) and recently divided (yellow) cells. Notice that bacteria are not affected by the smSRM imaging procedure and can grow and divide successfully when attached to the surface coated with chitosan.

To investigate the flatness of cells when immobilized in our surface-treated microfluidics chambers, we turned to 3D structure illumination microscopy (3D-SIM), a super-resolution microscopy method that allows for 3D, multi-color imaging of cells at twice the maximum resolution with conventional microscopies. We immobilized *B. subtilis* cells onto poly-L-lysine or chitosan-treated microfluidics chambers by following the protocol described above, and labelled membranes by using FM4-64. We evaluated whether cells were flat by: (1) drawing a line on the xy plane that passes through the cell poles and the center of the division septum (Figure 3Ai); (2) calculating the fluorescence signal across this line at different depths (z positions) (Figure 3Ai); and (3) measuring the distance between peaks at different z-positions. The plane corresponding to z = 0 is chosen as the axial plane that corresponds to the center of the open septum. Thus, in all cases line profiles show two peaks at z = 0, corresponding to the fluorescence intensity signals at the cell poles. Line profiles at higher or lower axial positions show a third peak at midcell corresponding to the fluorescence signal of the closing septum (Figure 3A, right panels). For simplicity, we define d_R_ as the distance between the right and the central peaks and and d_L_ as the distance between the left and the central peaks. On the one hand, when cells lay flat on the optical plane, the peaks are symmetrically located with respect to the center of the cell and this symmetry is conserved at different focal planes. In this case, d_R_ and d_L_ either decrease together or remain unchanged as we move to positive or negative z-positions (Figure 3Ai, right panel). In contrast, in inclined cells the location of peaks with respect to center of the cell is asymmetric, with peaks moving either to the left or to the right as the focal plane is approached. In other words, d_R_ increases and d_L_ decreases as we move to positive z-positions, with the contrary occurring when moving to negative z-positions (Figure 3Aii, right panel). Three planes of a representative 3D-SIM image of a *B. subtilis* cell immobilized on a microfluidics chamber are shown in Figure 3Bi, with three views of constant intensity level reconstructions shown in Figure 3Bii. In our immobilization conditions, most of cells (>90%) show symmetric profiles (Figure 3Biii) whose localization can be measured with high precision (<10 nm, as the SNR of the membrane signal is larger than 20 dB). These measurements are compatible with tilt angles <3 degrees (see Materials and Methods). On microfluidics chambers most cells are flat (>90%), independently of whether chitosan or poly-L-lysine were used to adhere cells.

Finally, we evaluated the ability of cells to grow and divide and the degree of movement of cells with respect to the surface in the time-scales required for smSRM imaging, as cell displacement cannot be corrected by the use of fiducial marks and would considerably compromise the reconstruction procedure. Cells immobilized on poly-L-lysine-coated support showed almost no movement (<5 nm in 10 min, see Figure S1) or change in cell morphology over the whole acquisition period, but were unable to grow or divide (30 min, Figure 3C). In contrast, cells attached to chitosan-coated surfaces were able to grow and divide with generation times of ∼10 hours while grown at 18°C with no exchange of medium and ∼75 min at 23°C under constant flow of growth medium, respectively (Figure 3D-E, Figure S2, Movies S1 and S2). These data are in good agreement with previously reported data (23.6 at 37°C, and ∼40 min at 30°C) [13], [38] and with our own observations under similar conditions in agarose pads (∼10 hours at 18°C, data not shown). Although cells are able to successfully grow in chitosan treated surfaces, we observed that daughter cells show a tendency to detach from the surface after one or two generations (i.e. one of the two daughter cells dissociates from the surface while the other remains attached) and the majority of cells does not remain attached for periods longer than 3 hours. When grown at 18°C, cells showed no detectable movement over a period of 10 min, making chitosan-immobilization compatible with smSRM imaging as long as acquisition times are not longer than a few tens of minutes. Another clear advantage of chitosan- over poly-L-Lysine-immobilization is that it allows for time-lapse smSRM imaging under physiological growth conditions (Figure 3D–E, Figure S2A, and Movies S1 and S2). Importantly, adhesion of cells to chitosan-treated surfaces does not affect their membrane potential (Figure S2B–D). Similar experiments were performed in *E. coli* with comparable results (data not shown).

### Automatic classification of cluster types and cell cycle stages

Next, we coupled our microfluidics-based, sequential, multi-color smSRM acquisition scheme described above to the automatic detection of protein cellular distributions and their classification in different cell cycle stages. This combination allowed for the determination of cell heterogeneity of protein distributions and structures at high-resolution, the analysis of protein behavior at the single cell level, and the establishment of mean localization properties in cell populations. To exemplify this methodology, we applied it here to the cell cycle-dependent cellular localization of SpoIIIE, a membrane-bound, double-stranded DNA motor responsible for chromosome translocation during sporulation and cell division in *B. subtilis*. SpoIIIE localizes in two types of structures: PALM-limited clusters are smaller than the localization precision of smSRM in our conditions (σ∼25 nm) and remain immobile during the acquisition time, whereas dynamic clusters are larger in size and represent single or multiple SpoIIIE proteins diffusing in the membrane [34]. The automatic classification of cluster types and cell cycle stages is divided in five sequential steps.

**Figure 4:**
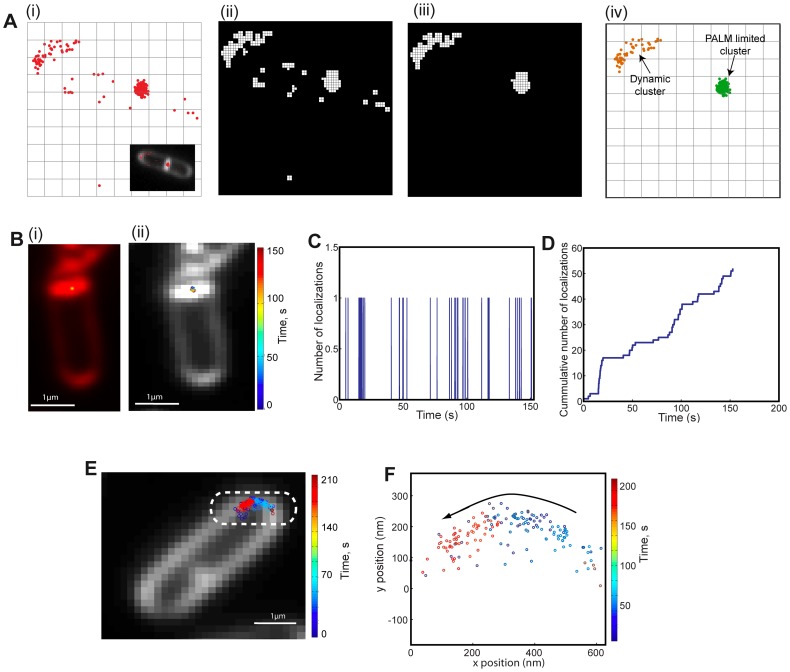
Automatic cluster detection and characterization. **A.** Scheme of the clusterization algorithm. (i) Red dots represent single-molecule localizations with black lines representing pixel boundaries of the smSRM image in the inset. (ii) Initially, the field of view is divided in virtual pixels of a size smaller than our localization precision (virtual pixel size is typically 5–10 nm) and a binary image of the localizations map is built (white pixels). (iii) The binary image is then analyzed, and independent objects are automatically detected and classified. Objects showing a minimum number of events (5 to 50) and area (>1 px) were further processed whereas the rest were discarded. (iv) Each cluster was classified depending on their size, number of events and trajectories. A dynamic (orange dots) and a PALM-limited cluster (green dots, see main text) were detected. Dots and pixel sizes were arbitrary modified for visualizing purposes. **B. PALM-limited clusters.** (i) smSRM reconstruction of the distribution of SpoIIIE-PA (SpoIIIE-mMaple), and (ii) a pointillist representation of a PALM-limited cluster in which single localizations are color-coded by time (complete time-series). **C–D. Analysis of single-molecule detections in a typical cluster. C.** Number of events detected in the PALM-limited cluster shown in B are plotted as a function of time. Single, non-overlapping localization events are detected and dark times between events are longer than average emission times, consistent with each photo-activated protein being imaged with no overlapping between events. **D.** Cumulative number of events detected as a function of time in the PALM-limited cluster shown in B. This trace shows that photo-activation rates are in average homogeneous during acquisition. **E–F Characterization of dynamic clusters. E.** A pointillist representation of a dynamic cluster (highlighted by a dotted line) in which single localizations are color-coded by time (complete time-series). Here, localization events spread over several pixels and follow a path along the cell pole. **F.** Representative trajectories generated from tracking the motion of single-localizations identified in panel E (color coded by time of detection: from latest to earliest red, green and blue).

First, an approximation of cell contours was obtained by using a modified version of microbeTracker [39] on DNA images, as fluorescence signal from nucleoids do not overlap even if cells are physically in contact (see below and Materials and Methods). The localizations found in the interior of the cell contour were further analyzed by using an automatic clustering algorithm. This algorithm consisted of the following steps: (1) the localizations in a region of interest (ROI) containing the cell contour were isolated (Figure 4A-i); (2) a new binary image was built in which pixels containing one or more localizations have a value of one, whilst those with no localization remain with a value of zero. The virtual pixel size in this image was chosen to be smaller (typically 5–10 nm) than the localization precision (∼25 nm) (Figure 4A-ii); (3) Binary objects were automatically segmented by finding the connected components in the binary image (using 8-connected pixels) (Figure 4A-ii); (4) Only binary objects containing more than a minimum number of localizations (typically 5–50, depending on fluorophore photo-physics and acquisition conditions) and with an area larger than one virtual pixel were kept for further processing (Figure 4A-iii); (5) Objects were separately classified and the list of localizations contained in that object were found; (6) To prevent large, sparse clusters from being artificially split into smaller ones, the area surrounding each cluster (C_i_) was probed to test whether other fluorescent events or close-by clusters could be connected to C_i_. This was achieved by increasing the radius of the search area by small steps (∼10–50 nm) around the cluster C_i_. If the number of events detected in the enlarged area increased by more than 5%, all the events were connected together to form a single cluster C_i_ and the search proceeded to the next area. However, if the variation in the number of events was lower than 5%, the search was stopped and another cluster C_i+1_ was analyzed. In the example in Figure 4A, two clusters were identified (Figure 4A-iv).

Second, each cluster was classified depending on its size, and its properties were analyzed by resorting to the single-molecule information contained in the times and coordinates of PALM-localizations composing the cluster. From the coordinates of PALM localizations, we measured the cluster sizes (full width at half maximum, or FWHM) and found that PALM-limited clusters are rather small (∼45 nm FWHM), while dynamic clusters can be several μm in size (Figure 4B and 4E). The dynamics of different clusters can be visualized by color-coding their single-molecule localizations by the time at which each localization was acquired. PALM-limited clusters are stable over the whole acquisition time (Figure 4B), while dynamic clusters represent proteins or groups of proteins that move during the acquisition (Figure 4E). To further characterize the clusters composition, we analyzed the number of localizations as a function of time for each individual cluster (Figure 4C) and the cumulative number of localizations (Figure 4D). From the former, it is possible to determine the average time that fluorescent proteins within a cluster are ‘on’ (actively emitting) versus the time they are ‘off’ (non photo-converted or in a dark state), a measure of the duty cycle of the fluorophore [40] and an indication of the single-molecule character of the acquisition process. For both PALM-limited and dynamic clusters, the frequency of photo-activations and the mean duty cycle correspond to well-spaced single-localizations (Figure 4C–D). These measures are important to certify that only one single-molecule is detected per diffraction-limited spot and become essential to characterize the quality of smSRM acquisition and quantify the number of events composing a specific cluster (proportional to the number of molecules). From the cumulative number of localizations, instead, it is possible to estimate the number of molecules in a cluster [41], [42], but this approach requires photo-activatable proteins displaying little or no blinking behavior.

When the movement of a cluster is slow with respect to the frame acquisition time (typically 30–50 ms), the single-molecules detected at different times during the movement of the cluster (Figure 4F) can be used to characterize the dynamics of the center of mass of the cluster. In this case, the type of diffusion (e.g. confined, brownian, etc), and the average diffusional properties can be calculated. As these measures are performed on single clusters, these data could also be used to identify different dynamical species [43], [44].

**Figure 5:**
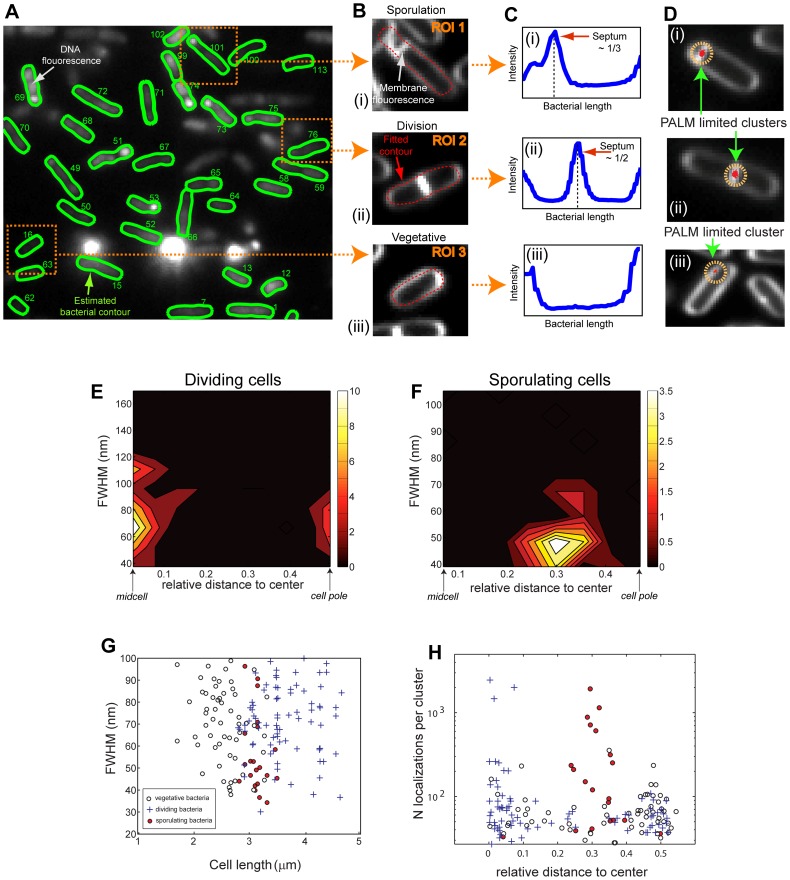
Automatic cell detection, sorting, classification, and cluster statistics. **A. Cell detection.** Chromosomal DNA stained with SYTOX green was imaged, cells were detected, and contours were calculated using a modified version of MicrobeTracker [39]. For each detected bacterium, the position of the cell and the points delimiting its contour were saved and fed to our PALMcbs software. **B–C. Contour refinement and automatic cell sorting. B.** Since chromosomes were naturally confined within the cell, the contour calculated with microbeTracker was often too small to encompass the whole membrane and required enlargement. Using the previously detected contour as a seed, the contours were recalculated by fitting it to the membrane fluorescence signal. **C.** A cell sorting procedure was used to classify cells according to the three growing states detectable in our experimental conditions. The presence of a septum was first assessed by plotting the fluorescence intensity profile of the membrane along the cell length. Depending on whether a septum was detected and on its position, each bacterium was automatically classified as sporulating, dividing or vegetative/pre-divisional (ROI 1, 2 and 3 respectively in panel B). **D. Cluster detection and classification.** First, single molecule events obtained from all frames are plotted together (red dots) and over-imposed on the membrane stain image (white). Next, the distribution of fluorescent events was analyzed as described in Figure 3 in order to automatically detect, classify and characterize clusters. **E–F. Distribution of SpoIIIE-mMaple PALM-limited cluster sizes in a population of (E) dividing and (F) sporulating bacteria.** The sizes of the detect clusters (FWHM, full width at half maximum) is plotted against the axial distance from the center of the bacteria. The total number of clusters detected is color-coded according to the color bar (right). (E) In dividing cells, clusters localize to division septa and to cell poles to a lower degree, and have a broad distribution with an average size of ∼65 nm. (F) In sporulating cells, clusters specifically localize to the sporulation septum and have a narrow distribution with a mean at ∼45 nm. **G. SpoIIIE-mMaple PALM-limited cluster sizes as a function of cell cycle stage and bacterial length.** The size of detected clusters was plotted against the bacterial length (in the axial direction) and classified depending on its cell cycle stage. Vegetative/pre-divisional cells tend to be small (2.5±0.5 μm) and show clusters of 65±15 nm in size, whilst dividing cells are considerably larger (4±0.6 μm) and display similar cluster sizes (65±18 nm). Interestingly, sporulating cells have a narrower size distribution (3.1±0.2 μm) and present smaller clusters (45±8 nm). **H. Composition and distribution of SpoIIIE-mMaple PALM-limited clusters as a function of cell cycle stage.** The number of localizations per cluster was plotted as a function of their relative axial distance to the center of the cell. Clusters in sporulating cells tend to have a larger number of localizations than clusters in dividing or vegetative/pre-divisional states.

Third, we classified cells in order to obtain mean localization statistics for each cluster type as a function of the cell cycle. Masks obtained from DNA images were used as a first approximation of the cell contour (seed, Figure 5A). Since chromosomes are confined within the cell, contours calculated with microbeTracker were often too small to encompass the whole membrane and required enlargement. Thus, contours were optimized by using an automated procedure that minimized the distance between the points defining the contour and the membrane signal of the corresponding cell (Figure 5B). Next, a cell sorting procedure was used to classify bacteria according to: vegetative/pre-divisional, dividing or sporulating cell cycle stages (Figure 5Bi-iii). The presence of a septum was first assessed by plotting the fluorescence intensity profile of the membrane along the cell length (Figure 5C). If no septum was detected, the bacterium was automatically classified as vegetative/pre-divisional (Figure 5C-iii). Otherwise, the cell was divided into three compartments of equal length. If the septum was found in the middle one, the cell state was classified as a cell undergoing division (Figure 5C-ii). Finally, if the septum was found in one of the two other compartments, the bacterium state was classified as a sporulating cell (Figure 5C-i). Automatic classifications were manually verified to prevent any errors in the interpretation of data due to cell misclassification (misclassification rate was typically ∼5% but strongly depended on the extent of cell packing).

Fourth, at the end of this automatic cluster and cell classification process, each single-molecule localization was assigned to a specific cluster type and cell cycle stage (Figure 5D-i, ii) permitting the study of cell-to-cell heterogeneities and mean population properties. As an example, we investigated the mean localization properties of SpoIIIE clusters by constructing probability distribution maps based on the sizes and distributions of PALM-limited clusters for each cell cycle stage. For instance, SpoIIIE clusters, ∼70 nm FWHM in size, localized to the center of the symmetric septum in cells undergoing cell division, with a small minority of cells showing clusters at the cell pole (Figure 5E). In contrast, SpoIIIE assembled in smaller clusters, ∼40 nm in size, which strongly localized to the center of sporulation septa during asymmetric septation (Figure 5F). Next, we plotted the distribution of cluster sizes as a function of cell length for each cell cycle stage (Figure 5G). Vegetative/pre-divisional cells have typical sizes of 2.5±.5 μm, cells undergoing division are typically 4±1 μm, while sporulating cells have a narrower length distribution of 3.1±0.2 μm. Interestingly, the size of PALM-limited SpoIIIE clusters is not only independent of cell cycle stage but also of cell length (Figure 5G). These results are consistent with previous measurements indicating that SpoIIIE is able to localize as PALM-limited clusters in pre-divisional cells before septum constriction, and that the size of translocating SpoIIIE clusters is the same in divisional or sporulating cells [34].

Finally, we computed the distribution of number of localizations in PALM-limited clusters as a function of their cellular localization and cell cycle stage (Figure 5H). The number of localizations in a cluster is proportional to the absolute number of molecules, but an accurate conversion is difficult as it requires a proper characterization of the photo-physical behavior of the PA protein in the cellular environment [41], [42]. We observed that SpoIIIE clusters have the highest number of localizations in sporulating cells when located on the asymmetric septum, have a decreased number of localizations on symmetric septa, and the lowest number of localizations when found in vegetative/pre-divisional cells. These results are consistent with previous observations, and with the main biological function of SpoIIIE being the translocation of DNA during *B. subtilis* sporulation, with a minor role during vegetative cell division [34].

## Discussion

In this study, we developed a micro-fluidics device combined with sequential injection of fluorophores and automatic cell and cluster detection that is ideally suited for the application of super-resolution microscopy to bacteria. Immobilization of bacterial cultures in thin agarose pads has been used in the past to perform super-resolution imaging [22], [23], [24], [25], [26]. However, as we showed in this study, this approach presents several important drawbacks: (1) cells often move over time with respect to fiducial marks, and different regions of the agarose pad move relative to each other; (2) agarose shows increased levels of background, degrading the precision of localization; (3) the roughness of the agarose surface makes cells often tilted with respect to the optical plane, resulting in the imaging of different planes in different bacteria; (4) increased background levels due to bleed-through between channels can lead to false positive localizations and lower localization precision; (5) agarose desiccation and the lack of a renewable medium lead to stress, thus preventing studies in live bacteria over long timescales (∼h). Some of these limitations can be overcome by hybrid microfluidics devices combining agarose and a custom flow chamber avoiding desiccation and permitting medium exchange [18], [21]. These methods, however, only address the last problem aforementioned. Micro-scale microfluidics have widely been used in eukaryotic cell biology [45], [46], and recently in bacteria [17], but these networks do not provide the long-term stability required for smSRM.

Our custom microfluidics device is simple to build and implement, can be easily adapted to different experimental requirements, is relatively inexpensive, and does not require micro-fabrication capabilities. In addition, lateral drift is homogeneous over the sample plane and can be corrected with nanometer precision even after tens of minutes of continuous illumination, unlike what is observed with agarose-based immobilization strategies. Cells immobilized on poly-L-lysine surfaces show undetectable movement with respect to fiducial markers, a requirement that is essential for building a proper, drift-corrected smSRM image from thousands of single-molecule localizations spread over minutes. Poly-L-lysine immobilization may, however, produce growth artifacts [47], and therefore an immobilization procedure without this deleterious effect was desirable. Here, we used chitosan, a linear polysaccharide that permits the stable surface immobilization of *B. subtilis*. Chitosan-treated surfaces are highly regular, as measured by wet-seec (wet surface-enhanced ellipsometric contrast microscopy) [48], and still allow for bacterial growth, motility and cell division to proceed. Other bacteria, such as *Escherichia coli* (data not shown) and *Myxococcus Xanthus* [37], [48], also adhere well to chitosan-functionalized surfaces and show normal motility and growth behaviors. In chitosan-functionalized surfaces, the lateral movement of fiducial marks was homogeneous over the imaging area and could be used to correct for drift with a resolution of ∼7 nm in the entire field of view. In addition, cells remained immobile over minutes, allowing for the monitoring of dynamical behaviors of proteins or complexes in the 1 s-5 min timescale, and the acquisition of super-resolution images of structures showing slow dynamics (∼tens of min). As cells grow and divide normally on chitosan surfaces with a rate that depends on growth medium, time-lapse smSRM imaging can be tailored to follow structural changes at slow timescales (few minutes to hours), provided that only a fraction of fluorophores are photo-activated during the acquisition of each smSRM image.

In addition to its advantages for smSRM microscopy, the methodology presented will be useful for a range of advanced fluorescence microscopy methods, such as 3D structured illumination microscopy [34], [49], [50], two-photon scanning fluorescence correlation spectroscopy methods (e.g. Number and Brightness analysis, or N&B) [51], [52], total internal reflection microscopy (TIRM) [53], or single-particle counting or counting experiments (e.g. slim field microscopy, or SFM) [16], [54]. The considerably lower background, higher stability, and lower fluorescence bleed-through afforded by this method is fundamental to all these technologies. In addition, the increased surface flatness is key for 2-photon methods due to their reduced depth of field, and to TIRM, as otherwise the variable thickness that separates coverslip from specimen makes the application of this method difficult. Finally, both single-molecule TIRM and SFM depend on the detection of single or a small set of fluorescent probes, thus the reduced fluorescence background and bleed-through becomes particularly important.

Cell cycle and cell-to-cell changes in stochastic transcription levels can lead to large heterogeneities in the structure and composition of molecular complexes [55]. Here, we addressed these heterogeneities by classifying cells in their different cell cycle stages and analyzing the composition, dynamics and cellular localization of two types of SpoIIIE complexes (PALM-limited and dynamic clusters). This approach allowed us to study the heterogeneities and average properties, such as size, relative number of molecules, and cellular localization, for each kind of complex on different stages of the cell cycle. We found that SpoIIIE strongly localizes in single, static clusters (∼ 45 nm FWHM) to the center of the asymmetric septum in sporulating cells, and to the symmetric septum (to a minor extent to the cell pole) in dividing cells. Interestingly, these clusters have a size that is mostly independent of cell cycle stage or cell length, but have a number of localizations (proportional to the number of molecules) that strongly depends on the stage of the cell cycle. This example shows the importance of classification of heterogeneous samples for drawing valid quantitative conclusions from smSRM imaging.

In summary, we described a flexible and versatile microfluidics device coupled to a sequential injection and imaging scheme, and automatic classification of cell cycle stage and type of cluster. This approach may be applied to study the architecture, composition and dynamics of single proteins or complex molecular machines in live bacterial cells, such as the flagellar motor, the conjugation/transformation machinery, or the division apparatus by super-resolution or advanced microscopy methods with a larger degree of long-term stability, and in a more controlled environment.

## Materials and Methods

### Strains and Cell Culture

SpoIIIE-PA was constructed by replacing the SpoIIIE gene of *B. subtilis* PY79 at its native location by a SpoIIIE-mMaple (used for PALM experiments on micro-fluidics chambers) or a SpoIIIE-eosFP (used for PALM experiments on agarose pads) fusion under the endogenous promoter [34]. These fusion strains no longer contained the wild-type copy of SpoIIIE and relied on the tagged protein for function. Fusions were fully functional, as cells producing the fusion protein were able to grow and sporulate as well as cells producing the unmodified DNA translocase. Cells were cultured as described elsewhere [34]. Briefly, after overnight culture in a solid plate, a single colony was serially diluted and grown overnight at 30°C in 20% Luria-Bertani medium (LB20%). The next morning exponentially growing cultures were diluted to OD ∼0.05 in LB20% and incubated at 37°C until reaching OD ∼0.6. Finally, cells were centrifuged, pelleted, and resuspended in pre-warmed sporulation medium [56]. Cells were incubated for 2 h in sporulation medium before imaging. DNA was stained with 7.5 nM of SYTOX green (Invitrogen, France).

### smSRM experiments on agarose pads

A double-side adhesive o-ring (1 mm thick, inner diameter 1.3 cm, outer diameter 2 cm, P18179, Invitrogen) was placed on a coverslip. 60 µl of 2.4% melted agarose (A4804, Sigma, France, diluted in PBS, melted at 90°C) were spread on the centre of the coverslip, which was then dried for 12 minutes in a sterile vertical laminar flow-hood. The high concentration of agarose was used to improve the stability of the pad during smSRM experiments. The agarose was melted slowly to prevent high auto-fluorescence background during smSRM experiments. In the meantime, cells were stained by adding 5 µl of a 0.3 µM solution of membrane dye (FM4-64, Invitrogen, France) to 1 ml of bacterial suspension. After five minutes incubation avoiding light exposure, a culture aliquot was spun down in a bench centrifuge at room temperature and 4000 rpm for 3 minutes, the supernatant discarded and the pellet resuspended in 60 µl filtered sporulation medium. Fluorescent beads (40 nm, T8860 Invitrogen or 100 nm beads, T7279, Invitrogen) were added to the suspension and 3 μl of the bacterial solution was pipetted onto agarose pad. The pad was then sealed with a clean coverslip and kept in the dark for three minutes to allow for spreading of the bacteria over the surface. The sample was finally fixed into an Attofluor cell (Invitrogen) to reduce agarose pad motion during microscopy.

### smSRM on microfluidics chambers

Holes in coverslips for inlets and outlets as well as channels in nescofilm masks were performed with a CO_2_ laser cutter (Thermoflan, France). Each channel was filled with 0.01% (w/v) poly-L-Lysine (P8920, Sigma) or 0.015% (w/v) chitosan, a linear polysaccharide composed of randomly distributed β -(1–4)-linked D -glucosamine (deacetylated unit) and N -acetyl- D -glucosamine (acetylated unit) (C3646, Sigma), and incubated for 5 min at room temperature, then rinsed sequentially with water and sporulation medium. A bacterial resuspension containing 40 nm fluorescent beads (Invitrogen, France) used as fiducial marks for drift correction was injected and incubated onto the poly-L-lysine- or chitosan-coated surface for 5 min. A high flow force (∼200 μL/s of sporulation medium) was applied to flatten cells against the surface. When using poly-L-lysing-coated surfaces, each experiment was performed in a new channel, and measurements were performed within the first 15 min after injection of cells into the channel to avoid any possible surface-immobilization effect.

### smSRM instrumentation and imaging

Imaging was performed as described elsewhere [34]. Briefly, experiments were performed in a modified Nikon Eclipse Ti-S inverted microscope equipped with a 100× Plan-Apo oil-immersion objective (NA  = 1.4). Four lasers with excitation wavelengths of 405 nm (Vortran Technology, Sacramento, USA), 488 nm (Coherent, Inc, Santa Clara, USA), 532 nm (Laser Quantum, Cheshire, UK) and 561 nm (Coherent, Inc, Santa Clara, USA) were employed and emission fluorescence collected in an emCCD camera (Andor Ixon 897, Ireland). In agarose-pad experiments, SpoIIIE-PA was imaged by exciting with a 532 nm laser and collecting the fluorescence signal through a ET605/70 emission filter (Chroma, Bellows Falls, USA), while FM4-64 was excited at 488 nm and its fluorescence emission collected by a ET700/75 m emission filter (Chroma, Bellows Falls, USA). For experiments in microfluidics chambers, SYTOX was excited at 488 nm, while SpoIIIE-PA and FM4-64 were excited at 561 nm. In all cases, the fluorescence signal was filtered through a ET605/70 filter to avoid chromatic aberrations. Excitation and emission signals were separated by a single four-band dichroic filter: zt/405/488/561/633rpc or zt/405/488/532/633rpc, depending on whether the 561 nm or the 532 nm laser was used (Chroma, Bellows Falls, USA). Pixel size was 110 nm. Acquisition software controlling lasers, filter wheels, and camera were homemade using LabView 2010 (National Instruments, France).

An active autofocus system is essential for smSRM imaging, as drift in the optical axis can reach hundreds of nanometers in the timescales used for a typical smSRM experiment (∼10 min). To avoid loss of focus during PALM acquisition, an active feedback autofocus system was home built. This system locked the focal plane position with a stability of ∼10 nm over hours (data not shown). In a separate path from the other four lasers, a linearly polarized 1064 nm infrared (IR) beam from an Ytterbium fiber laser (YLM5-1064-LP, IPG Photonics Laser, Oxford, USA) was expanded by using two lenses in a telescopic configuration and passed through an optical separator formed by a polarized beam splitter (PBS) and a quarter wave plate (QWP). The beam was directed towards the objective lens by a dichroic mirror. Depending on the sample and the position of the plane imaged by the objective, the distance between the lenses composing the telescope was modified to ensure that the IR beam was always focused at the glass/water interface. Part of the IR beam was reflected by the sample, collected by the objective and redirected towards the PBS following the same path than the incident beam. Due to a change in polarization introduced by reflection at right angles, the reflected beam was redirected by the PBS and imaged on a CCD detector. A half-wave plate (HWP) before the PBS was used to manually adjust the intensity of the incident beam. Control software for the autofocus was written in LabView 2009, using the PID and Fuzzy Logic Toolkits. A feedback loop between the CCD detector (C2) and the piezo stage (PZ) was used to make sure that the sample remained in focus at all times during the PALM acquisition. At the beginning of each experiment, a calibration is carried out to ensure that the intensity of the IR reflection varies linearly over a course of ∼600 nm around the plane imaged by the objective. When the acquisition starts, the intensity of the IR reflection is used as reference and axial drift was corrected by adjusting the position of the objective.

Misalignment and chromatic aberrations between images obtained at different times and using different filters were corrected using the positions of fluorescent beads scattered over the field of view. For each two sets of images, the mean positions (X_i_ and Y_i_) and the localization errors (σ_Xi_ and σ_Yi_) were calculated for each bead. In agarose pads, for a given ROI enclosing the cell of interest, a minimum of seven proximal beads were automatically selected and a second-order polynomial transformation T was inferred from their positions. This transformation allowed for the correction of image translation and rotation as well as variation in magnification. The quality of the alignment was assessed by comparing the variation in the bead positions between two images, before and after applying the correction (see Figure 1D and E). Typically, a shift of several pixels in bead positions (ΔY, Figure 1D) may be measured between images taken in different colors when no correction is applied. After applying the polynomial transformation, both ΔX and ΔY values fell below 0.1 pixel (i.e. 11.5 nm for our pixel size), illustrating the quality of the alignment correction.

Lateral drift correction in agarose pads was performed as follows: (1) for each cell, the trajectories of seven close-by beads were chosen and the mean trajectory was calculated by averaging the seven trajectories; (2) The quality of the drift correction was estimated by subtracting the mean trajectory to all the trajectories and calculating the standard deviations along x and y for the beads used for correction; (3) when the standard deviation of the seven beads was acceptable (<10 nm), the coordinates detected inside the cell were corrected by using the mean trajectory. The same procedure was applied in microfluidics chambers but a smaller number of beads can be used (>4 are enough to obtain a nanometer correction) and the same correction was applied to all cells in the field of view without compromising the quality of the correction.

### 3D structured illumination microscopy

Samples for 3D-SIM experiments were prepared as for smSRM experiments (see above). 3D-SIM imaging was performed on an OMX V3 microscope (Applied Precision, USA) using 561 nm laser and the corresponding standard drawer. Reconstruction and alignment of 3D-SIM images was performed using softWoRx v 5.0 (Applied Precision, USA.). After calibration, the measured resolution of our OMX microscope was 120 nm for FM4-64. Pixel sizes were 40 nm in the lateral direction and 125 nm in the axial direction.

The signal-to-noise ratio of FM4-64 in 3D-SIM experiments was at least 20 dB, which translates into a localization precision of the center of the membrane of at least 8 nm [57]. This precision of localization can be used to analytically estimate from geometrical considerations the minimal tilt angle that can be detected with the procedure described in Figure 3A. For a localization precision of 8 nm, the minimal angle that can be detected is 3.3 degrees.

### Software for image analysis

Program development and image analysis were performed using MATLAB (MathWorks, Natick, USA), and is available upon request.

## Supporting Information

Figure S1 **Quantification of bacterial movement on a poly-L-lysine treated surface.** **A.** Bright field image of *B. subtilis* cells in a microfluidics chamber coated with poly-L-lysine (i) immediately after injection and (ii) 40 min after imaging. Yellow dotted line across bacteria in (i) and (ii) indicates the direction used to calculate intensity profiles shown in panel B. Encircled numbers indicate the bacteria employed as representative examples for movement estimation in the following panels. **B.** Line scan of light intensity across the two bacteria indicated in A. The intensity profiles at different imaging times (0, 10, 20, 30 and 40 min) are represented (see color coded time in the inset of the plot). Dotted ellipses indicate the zoomed areas shown in panel C. **C.** To estimate the bacterial edge with nanometer precision, a Gaussian-based multi-peak fitting function was employed (Origin Pro 8, Northampton, MA, USA). From the fitting of the multi-peak function (red solid line) to the experimental data (black dots) the lateral coordinates of the edge of each bacteria at each imaging time where obtained (x_l,j,t_ and x_r,j,t_ where superscripts r and l indicate whether the peak coordinate corresponds to the left or right peaks, j is an index that indicates the cell number, and t is the imaging time). The evolution of the fitted profiles obtained at different imaging times (blue and green solid lines) is represented. The raw data is not represented for clarity purposes. The x-coordinate of the center of gravity of each bacterium at time t was estimated as X_j,t_ =  (x_l,j,t_ – x_r,j,t_)/2. **D.** Lateral displacements between cells were estimated by calculating the absolute change in the distance between the center of two given bacteria from time  = 0 to time t as Ψ (t)  =  | (X_j,t_ – X_j+1,t_)/2– (X_j,0_ – X_j+1,0_)/2|. To obtain average values of Ψ (t), we repeated this process for five couples of bacteria. Black dots and error bars represent the mean value of Ψ and its dispersion over time. Grey solid line is a guide to the eye. Cells typically move less than 10 nm in 30 min.
(TIF)

Figure S2 ***B. subtilis*** **cells can grow and divide on chitosan-treated surfaces and their membrane integrity is not affected.** **A.** Exponentially growing cells (OD ∼0.3) were injected into a chitosan-treated microfluidics chamber, and attached to the surface as described in Figure 2B. Time-lapse bright field imaging was performed to determine wether cells were able to grow and divide while attached to chitosan-treated surfaces. Experiments were performed at 23°C while renewing the growing medium (5 µL/min of LB20%). Color-coded arrows indicate elongating (green), pre-divisional (orange) and recently divided (yellow) cells. **B–C.** A culture of *B. subtilis* cells was grown to OD ∼ 0.3 and an aliquot of 1 ml was incubated with 30 μM DiOC_2_ (3) for 30 min according to the manufacturer instructions (Invitrogen, France) and then injected into a microfluidics chamber coated with chitosan or deposited into an agarose pad. Bright field images of cells attached to a (**B–i**) chitosan treated-surface or **(C–i)** in an agarose pad after 20 min incubation in the flow cell or pad, respectively. Cells stained with the fluorescent dye DiOC_2_ (3) were excited at 488 nm, and imaged at (**ii**) 525 nm and (**iii**) 605 nm. Fluorescence intensity and contrast in panels ii and iii were fixed to identical values to facilitate visual comparison of fluorescence intensity in both types of surfaces. Images were pseudo-colored for illustrative purposes. **D.** Quantification of the membrane potential by monitoring the relative red/green fluorescence ratio in cells stained by DiOC_2_ (3) on chitosan-treated surfaces or in agarose pads (green columns). As a negative control, we tested the effect of the respiration uncoupler Carbonyl Cyanide m-Chloro Phenyl hydrazone (CCCP) which collapses the membrane potential. After imaging live bacteria in presence of DiOC_2_ (3), a solution of 10 μM CCCP was injected into the microfluidics chamber. Imaging of depolarized cells was performed after 10 min incubation with CCCP (orange column). The red/green fluorescence emission ratio was identical in chitosan-treated microfluidics chambers or in agarose pads, and increased when cell membranes were depolarized using CCCP. These data are consistent with the membrane potential of *B. subtilis* cells not being affected by their adhesion to chitosan-treated surfaces.
(TIF)

Movie S1 Time-lapse bright field imaging of *B. subtilis* cells growing in chitosan treated surfaces at 23°C with media injection (5 µl/min of LB20%). Images were acquired each 2 min.
(AVI)

Movie S2 Time-lapse bright field imaging of *B. subtilis* cells growing in chitosan treated surfaces at 23°C with media injection (5 µl/min of LB20%). Images were acquired each 2 min.
(AVI)
