# Supplementary figures and images for: Super-Resolution Imaging of Bacteria in a Microfluidics Device
Source: PLoS One. 2013 Oct 16;8(10):e76268. doi: 10.1371/journal.pone.0076268 (PMC3797773; doi:10.1371/journal.pone.0076268)

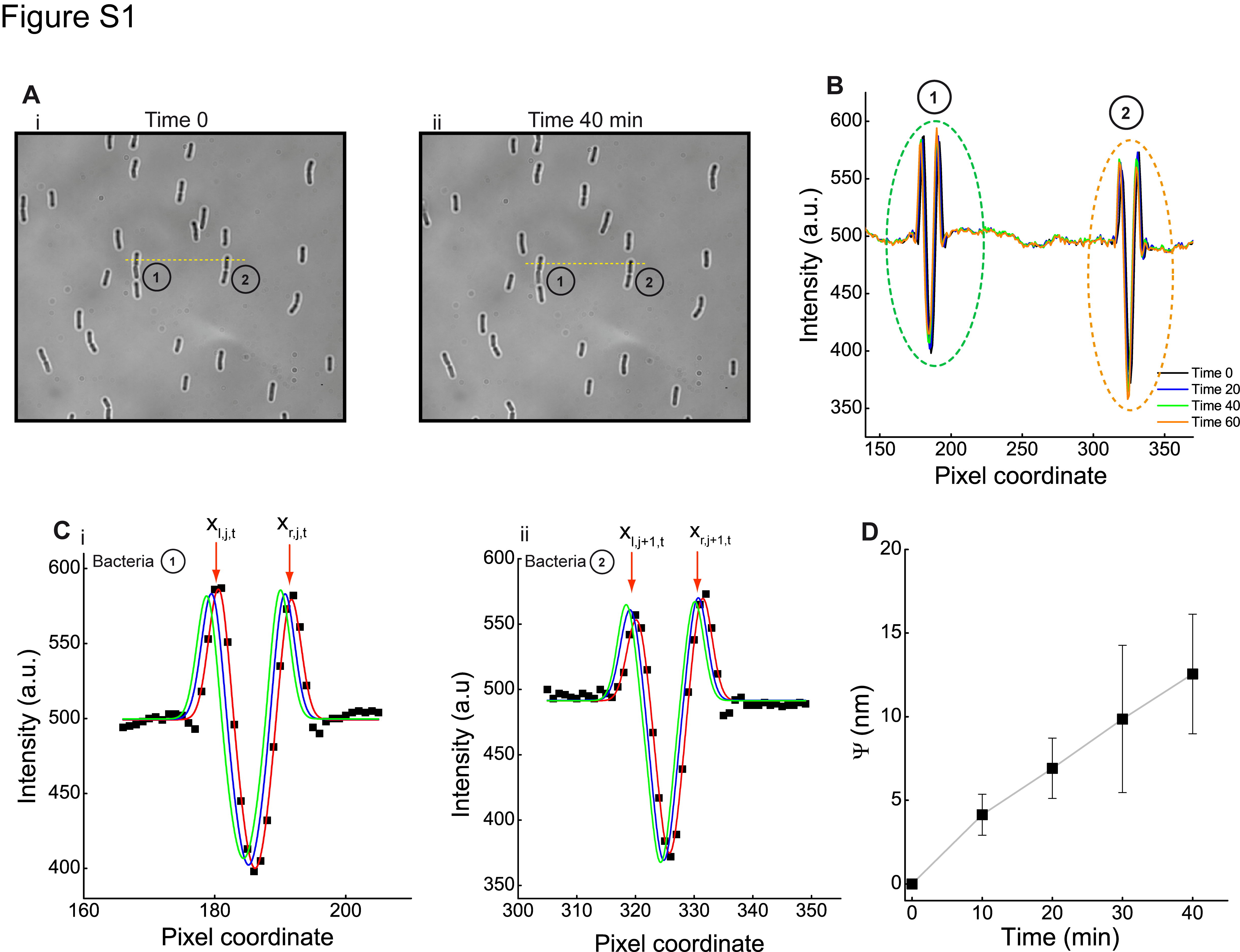

Supplement: Figure S1 — Quantification of bacterial movement on a poly-L-lysine treated surface. A. Bright field image of B. subtilis cells in a microfluidics chamber coated with poly-L-lysine (i) immediately after injection and (ii) 40 min after imaging. Yellow dotted line across bacteria in (i) and (ii) indicates the direction used to calculate intensity profiles shown in panel B. Encircled numbers indicate the bacteria employed as representative examples for movement estimation in the following panels. B. Line scan of light intensity across the two bacteria indicated in A. The intensity profiles at different imaging times (0, 10, 20, 30 and 40 min) are represented (see color coded time in the inset of the plot). Dotted ellipses indicate the zoomed areas shown in panel C. C. To estimate the bacterial edge with nanometer precision, a Gaussian-based multi-peak fitting function was employed (Origin Pro 8, Northampton, MA, USA). From the fitting of the multi-peak function (red solid line) to the experimental data (black dots) the lateral coordinates of the edge of each bacteria at each imaging time where obtained (xl,j,t and xr,j,t where superscripts r and l indicate whether the peak coordinate corresponds to the left or right peaks, j is an index that indicates the cell number, and t is the imaging time). The evolution of the fitted profiles obtained at different imaging times (blue and green solid lines) is represented. The raw data is not represented for clarity purposes. The x-coordinate of the center of gravity of each bacterium at time t was estimated as Xj,t = (xl,j,t – xr,j,t)/2. D. Lateral displacements between cells were estimated by calculating the absolute change in the distance between the center of two given bacteria from time = 0 to time t as Ψ (t) = | (Xj,t – Xj+1,t)/2– (Xj,0 – Xj+1,0)/2|. To obtain average values of Ψ (t), we repeated this process for five couples of bacteria. Black dots and error bars represent the mean value of Ψ and its dispersion over time. Grey solid li [file pone.0076268.s001.tif]

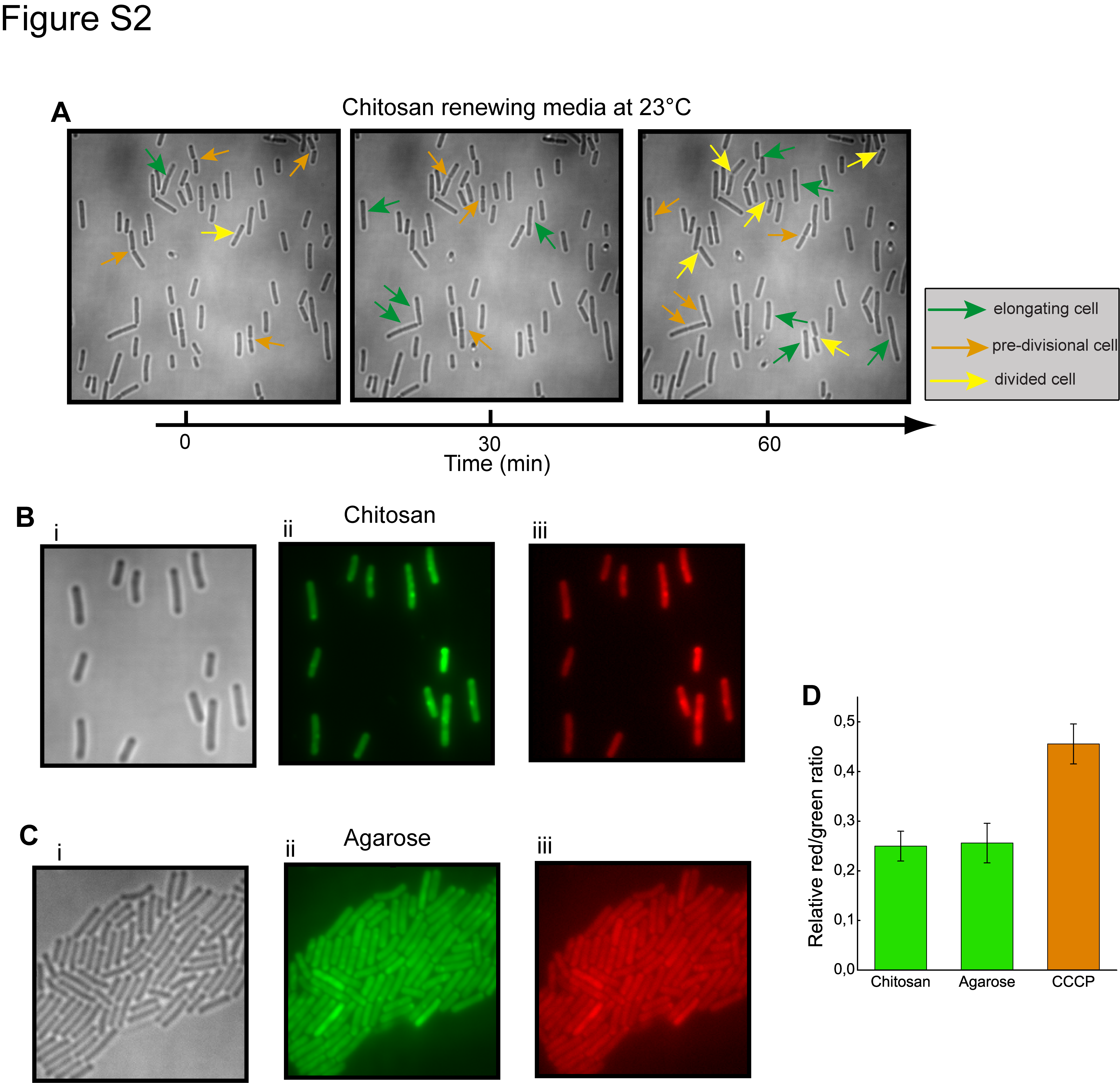

Supplement: Figure S2 — B. subtilis cells can grow and divide on chitosan-treated surfaces and their membrane integrity is not affected. A. Exponentially growing cells (OD ∼0.3) were injected into a chitosan-treated microfluidics chamber, and attached to the surface as described in Figure 2B. Time-lapse bright field imaging was performed to determine wether cells were able to grow and divide while attached to chitosan-treated surfaces. Experiments were performed at 23°C while renewing the growing medium (5 µL/min of LB20%). Color-coded arrows indicate elongating (green), pre-divisional (orange) and recently divided (yellow) cells. B–C. A culture of B. subtilis cells was grown to OD ∼ 0.3 and an aliquot of 1 ml was incubated with 30 μM DiOC2 (3) for 30 min according to the manufacturer instructions (Invitrogen, France) and then injected into a microfluidics chamber coated with chitosan or deposited into an agarose pad. Bright field images of cells attached to a (B–i) chitosan treated-surface or (C–i) in an agarose pad after 20 min incubation in the flow cell or pad, respectively. Cells stained with the fluorescent dye DiOC2 (3) were excited at 488 nm, and imaged at (ii) 525 nm and (iii) 605 nm. Fluorescence intensity and contrast in panels ii and iii were fixed to identical values to facilitate visual comparison of fluorescence intensity in both types of surfaces. Images were pseudo-colored for illustrative purposes. D. Quantification of the membrane potential by monitoring the relative red/green fluorescence ratio in cells stained by DiOC2 (3) on chitosan-treated surfaces or in agarose pads (green columns). As a negative control, we tested the effect of the respiration uncoupler Carbonyl Cyanide m-Chloro Phenyl hydrazone (CCCP) which collapses the membrane potential. After imaging live bacteria in presence of DiOC2 (3), a solution of 10 μM CCCP was injected into the microfluidics chamber. Imaging of depolarized cells was performed after 10 min incubation with CCCP (orange column). The red/ [file pone.0076268.s002.tif]
